# *Trichophyton indotineae Erg1*^Ala448Thr^ Strain Expressed Constitutively High Levels of Sterol 14-α Demethylase *Erg11B* mRNA, While Transporter *MDR3* and *Erg11A* mRNA Expression Was Induced After Addition of Short Chain Azoles

**DOI:** 10.3390/jof10110731

**Published:** 2024-10-22

**Authors:** Nadine Berstecher, Anke Burmester, Deborah Maria Gregersen, Jörg Tittelbach, Cornelia Wiegand

**Affiliations:** Department of Dermatology, Jena University Hospital, Friedrich Schiller University, D-07747 Jena, Germany

**Keywords:** ergosterol biosynthesis, sterol 14-α demethylase, *Erg11*, squalene epoxidase, *Erg1*, MDR transporter, major facilitator superfamily, *MFS1*, heat shock proteins, *HSF1*

## Abstract

*Trichophyton indotineae* is an emerging pathogen causing recalcitrant skin infections and exhibiting multiple resistances to azoles and allylamines. Squalene epoxidase *erg1*^Ala448Thr^ mutants often show association with azole resistance. RT-PCR gene expression analysis helps to elucidate the connection between ergosterol biosynthesis regulation and efflux control through the activation of multidrug resistance (MDR) and major facilitator superfamily (MFS1) transporters as well as heat shock proteins (HSP). Several *T. indotineae* isolates demonstrated a heat-dependent increase of *Erg11B* transcripts combined with downregulation of *Erg1*, suggesting a protective role for *Erg11B*. They also showed persistent upregulation of *MFS1.* The addition of fluconazole or voriconazole induced the expression of *Erg11A*, *MDR3* and, to a lesser extent, *Erg11B* and *Erg1*. The azole-resistant *erg1*^Ala448Thr^ mutant UKJ 476/21 exhibited exceptionally high transcript levels of sterol 14-αdemethylase *Erg11B*, combined with the inability of HSP60 and HSP90 to respond to increasing growth temperatures. Itraconazole demonstrated similar effects in a few *T. indotineae* isolates, but terbinafine did not enhance *Erg1* transcription at all. Overexpression of *Erg11B* may explain the multiple azole resistance phenotype, whereas *Erg11B* point mutations are not associated with resistance to azoles used for medical treatment.

## 1. Introduction

*Trichophyton indotineae* is an emerging pathogen that has developed multiple resistance mechanisms to antifungal agents such as azoles or allylamines [1,2,3]. Initially, the species was named *T. interdigitale* [1] due to the anthropophilic character of the pathogen and the unknown animal reservoir. However, ITS sequence comparisons within the species *T. mentagrophytes*/*T. interdigitale* complex showed a relation to zoophilic species of this complex [2]. Phylogenetic analyses based on complete genomes confirmed the basal lineage of the *T. indotineae* species strain D15P135 within the complex [4]. Later, isolates belonging to this subtype were renamed *T. indotineae* based also on the mating behavior of the isolates [5,6]. *T. indotineae* infections most commonly cause Tinea cruris or corporis in humans [7,8], while *T. interdigitale* is clinically typically associated with Tinea pedis [9].

Resistance to the allylamine terbinafine is mainly based on point mutations of the squalene epoxidase gene *Erg1,* which leads to exchanges of amino acids in binding positions of the antifungal compound in the protein [10]. *erg1*^Phe397Leu^ mutants represent the most common type of terbinafine-resistant isolate [1,3,5,6,7]. The *Trichophyton* *erg1*^Phe397Leu^ mutation corresponds to the baker’s yeast *erg1*^Phe402^ position that, when replaced with leucine, also confers resistance to terbinafine [11]. Interestingly, double mutants of *T. indotineae erg1*^Phe397Leu, Ala448Thr^ combine terbinafine resistance with fluconazole resistance [12,13,14]. Although some *erg1*^Ala448Thr^ single mutants show the multiple azole resistance phenotype [3,14,15], other *erg1*^Ala448Thr^ mutants react sensitively to azoles [15]. Therefore, the azole-resistant phenotype has to depend on more than one genetic factor. In *erg1* double mutants, the ratio of combined resistance to azoles and terbinafine is significantly increased [14], suggesting that double mutants evolved from the azole-resistant portion of *erg1*^Ala448Thr^ single mutants. The molecular mechanism behind the link between the *erg1* mutation and azole resistance has not yet been addressed.

*T. indotineae* is also an emerging pathogen of dermatomycosis in Germany [16]. To date, there is no evidence that local sources of infection play a significant role, and infections have been predominantly found in travelers or residents of foreign countries who started work or their studies in Germany [16]. The challenge in treatment of the infection is the insufficiency of focusing on species identification alone because *T. indotineae* isolates differ in their resistance patterns to terbinafine or specific azoles [15].

*T. indotineae* isolates also exhibit mutations in the sterol 14-α demethylase *Erg11B* gene [15]. In most of these isolates, an amino acid motif in *Erg11B* positions 441-444 is affected [15], which belongs to a fungal-specific loop of the sterol 14-α-demethylase [17]. Another mechanism for changing the azole resistance pattern is through an increase in *Erg11B* gene copies from one copy to up to seven copies of tandem repeats within a genome, leading to an increase in *Erg11B* transcript production [18]. Interestingly, the *T. indotineae* isolate TIMM20117 showed increased *Erg11B* transcript expression without gene amplification [18] in combination with a resistant phenotype. In *Trichophyton quinckeanum*, a zoophilic pathogen of mice, the point mutation in *Erg11A* correlates with a higher tolerance to itraconazole [19]. While no point mutations were found in *Erg11A* of *T. indotineae* isolates [15], no mutant *Erg11B* variants were detected in *T. quinckeanum* subtypes [19], suggesting that the roles of the two *Erg11* variants in both species could differ.

Another mechanism of fungal resistance depends on the expression of transporters belonging to the major facilitator superfamily (MFS) or the multidrug resistance (MDR) type. The latter belong to the ATP-binding cassette (ABC) containing transporters. Transporters of these classes are mainly responsible for the efflux of antifungal compounds in *Candida* [20]. The role of MDR and MFS transporters was further studied in *Trichophyton rubrum* [21], showing that *T. rubrum MDR3* expression mediates azole resistance. *MDR3* expression was also analyzed in *T. indotineae* strains with different itraconazole and voriconazole susceptibilities, demonstrating that *MDR3* is overexpressed in azole-resistant strains [18]. Nevertheless, *MDR3* disruption affects susceptibility to a low extent compared to the parental strains [18], which indicates that the behavior of the observed resistance phenotype is multifactorial.

This study investigates *T. indotineae* isolates from routine cases at the Department of Dermatology at Jena University Hospital, which were previously analyzed for antifungal resistance patterns as well as mutations within *Erg1* and *Erg11A* and *B genes* [15] for the expression of MDR and MFS transporter, ergosterol biosynthesis and heat shock protein genes.

## 2. Materials and Methods

### 2.1. Strains and Growth Conditions

*T. indotineae* strains from Jena University Hospital (UKJ) were stored as glycerol stocks at −80 °C and cultured on dermasel (Thermoscientific, Wesel, Germany) or Sabouraud dextrose (2% *w*/*v* dextrose, SDA) agar plates (Biomerieux, Marcy-l’Étoile, France) for a minimum of three weeks and up to eight weeks at room temperature. The cultivated plates were used to harvest spores for RNA isolation. In brief, the spores were suspended in 5 mL sterile, isotonic NaCl solution (9 g/L; Fresenius Kabi, Bad Homburg, Germany). The suspension was then filtered through a cell strainer with a mesh size of 40 µm (Greiner Bio-One, Kremsmünster, Austria) to remove mycelial debris. Spore concentration was estimated by counting spores using disposable counting chambers (type Neubauer improved; Carl Roth, Karlsruhe, Germany). Adjusted spore solutions were also plated on SDA agar to evaluate spore viability. For RNA isolation, 20 mL of SDA liquid medium was adjusted to a final concentration of 2 × 10^3^ cfu (colony forming units)/mL. The suspensions were transferred to sterile conical flasks of 100 mL volume and incubated at room temperature on a shaker at 120 rpm (rounds per minute) for six days. Three experimental series were performed. The first series was started in the summer months with daily peak temperatures of 37 °C and periodic cooling at night. The second series was carried out in a shaking water bath at a constant temperature of 37 °C. The last series was realized in the autumn and winter months at room temperatures between 20 to 25 °C.

### 2.2. Strain Evaluation

*T. indotineae* strain CBS 146726 (formerly UKJ 594/19) was analyzed for ITS region and squalene epoxidase *Erg1* gene sequences, as previously described [7]. For PCR, ITS primers were used as described before [3], and for *Erg1*, the primer pair TmSQLEF4 and TmSQLER4 was used for amplification, as described in [7]. Strains CBS 146726, CBS 146727 (formerly UKJ 1145/19), UKJ 888/20, UKJ 262/21, UKJ 476/21 and UKJ 1067/21 were analyzed for the DNA sequence information of the ITS region, *Erg1*, and the sterol 14-α demethylase gene fragments *Erg11A* and *Erg11B*, as previously described [15]. *T. indotineae* isolates UKJ 1985/21 and UKJ 1067/21 derived from the same 43-year-old patient with a sampling time difference of six months. During this time, the patient was systemically treated with terbinafine and additionally received topical treatment with ciclopirox olamine for a period of 12 weeks. Strain UKJ 579/22 caused tinea barbae in a 33-year-old male patient. Gene sequence information for ITS, *Erg1*, *Erg11A* and *Erg11B* for strains UKJ 1985/21 and UKJ 579/22 were deposited in GenBank Acc. No. PP537547-PP537548 and GenBank Acc. No. PP549424-PP549429. The strains, genes and GenBank Acc. Nos. of all isolates were listed in Table S1. The amino acid exchange of *Erg1* and *Erg11B* of each strain as well as inhibitory concentrations against terbinafine and itraconazole are summarized in Table S2. The majority of strains were analyzed previously [15].

### 2.3. Induction of Gene Expression with Antifungal Compounds

For the evaluation of the influence of antifungals on RNA expression, several compounds were added at the specified final concentration, which was below a strong inhibitory fungistatic or fungitoxic effect. Stock solutions were prepared in dimethyl sulfoxide (DMSO, Sigma Aldrich, St. Louis, MO, USA) for fluconazole (10 mg/mL [22], Sigma Aldrich), voriconazole (1 mg/mL [23], Sigma Aldrich), itraconazole (0.03 mg/mL [24], Sigma Aldrich) and terbinafine (0.08 mg/mL [25], Sigma Aldrich). Itraconazole concentrations were validated with an UV spectrophotometric method, as described [26], to minimize weigh errors. Working concentrations were adjusted to 10 µg/mL for fluconazole, 50 ng/mL for voriconazole, 1.5 ng/mL for itraconazole and 4.0 ng/mL for terbinafine in liquid SDA growth media. Spore suspension was added to a final concentration of 2 × 10^3^ cfu (colony forming units)/mL, as described above. The antifungal compounds were present throughout the growth phase of the fungal cultures.

### 2.4. RNA Isolation and Real-Time PCR

The mycelium was harvested using Miracloth filter screens (Calbiochem, San Diego, CA, USA) of approx. 5 cm^2^ size placed in small glass funnels. Residual liquid media was removed by manually squeezing the filters, and the mycelium mats were transferred immediately into a mortar pre-cooled with liquid nitrogen. The frozen mycelium was crushed under the addition of liquid nitrogen using a mortar and pestle. The cell debris were dissolved in one of the following lysis buffers, yielding similar RNA amounts: 0.5 mL RLT buffer (Qiagen, Hilden, Germany) containing 10 µL/mL 2-mercaptoethanol (freshly added before use). Alternatively, we used ELT lysis buffer containing 4 M guanidinium thiocyanate, 50 mM Tris-HCl pH7.5, 25 mM EDTA, 3% *v*/*v* Triton X100 [27] or ALT buffer containing 4 M guanidinium thiocyanate, 0.1 M sodium acetate pH 4.5, and 0.5% *w*/*v* lauryl sarcosine modified after a described prescription [28]. All probes were incubated for 3 min at 55 °C, and cell debris were precipitated using a centrifuge at 8000 rcf for 2 min at 20 °C. The supernatant of RLT and ELT probes was purified following the instructions of the Plant and Fungal Purification Mini kit (Qiagen). RNA of ALT supernatants was precipitated by adding 1/10 volume of 3 M sodium acetate pH 4.5 and 1/2 volume of 100% *v*/*v* ethanol before incubation on ice for 2 h. The probes were centrifuged at 8000 rcf for 5 min at 20 °C, and the pellets were resuspended in RLT buffer for purification, as described above. Purifications were carried out automatically using a Qiacube workstation (Qiagen) with an elution volume of 50 µL. For probes pretreated with ALT buffer, the elution step was repeated with an additional 50 µL of elution buffer due to the higher RNA yields. To avoid genomic DNA, all RNA probes were digested with DNase I kit (Thermo Fisher, Waltham, MA, USA) according to the manufacturer’s instructions. The RNA concentration of all probes was determined using the UV/Vis spectrometer OMEGA (BMG Labtech, Ortenberg, Germany) with the Lvis-plate for measuring small volumes of 2 µL at 260 nm. A total of 400 ng of RNA was transcribed into cDNA using the High-Capacity cDNA Reverse Transcription kit (Thermo Fisher), following the instructions. For quantitative real-time PCR, QuantiNova SYBR Green PCR kit (Qiagen) was used. The cDNA was diluted in yellow buffer (Qiagen) to a final concentration of 0.5 µg/mL. For a PCR reaction, 10 µL SYBR Green master mix was added to the forward and reverse primer at final concentrations of 500 nM and supplemented with PCR grade water to a final volume of 17 µL. Then, 3 µL of diluted cDNA was added. PCR was performed using qTOWER^3^G device (Analytik Jena, Jena, Germany) with the PCR protocol previously described [29].

### 2.5. Primer of Analyzed Genes and Expression Data Analysis

For transporter genes *MDR1*, *MDR2*, *MDR3* and *MFS1,* the nomenclature and primer sequences were adapted from primers developed for *T. rubrum* [21]. Actin (*Act1*) primers were used as control for a constitutive expressed gene, as described before [21]. Modifications were included if DNA sequences of the *T. indotineae* D15P135 genome [4] showed differences to *T. rubrum* primer sequences. Squalene epoxidase gene *Erg1* primers were used as described for qPCR [30]. For the sterol 14-α demethylase genes *Erg11A* and *Erg11B,* primers were developed using software tools (https://eurofinsgenomics.eu/en/ecom/tools/pcr-primer-design, accessed on 26 January 2022) from the primer manufacturer (Eurofins Genomics, Ebersberg, Germany). The primers for the heat shock genes *HSF1*, *HSP60* and *HSP90* were designed from primers for *T. rubrum* [31] after a comparison with the genomic information of *T. indotineae* D15P135 [4]. All primer sequences are listed in Table S3. The relative expression compared to untreated controls was calculated according to the primer efficiency-corrected algorithm using *Act1* as the housekeeping gene reference [32]. Non-detects were set to the CT maximum 40 as described [33]. To display the expression levels of all isolates maintained under untreated conditions, the expression levels were normalized against the housekeeping *Act1* gene using 2^−∆∆CT^ method [34]. RT-PCR data were obtained from two biological and two technical replicates for each condition depending on different temperatures or antifungal induction. Error bars in graphical images represent differences in biological and technical replicates.

### 2.6. Graphical Image Preparation and Statistical Analyses

Graphical images were prepared using the OriginPro 2019 (Origin-Lab Corporation, Northampton, MA, USA) software, while statistical analysis was performed with IBM SPSS Statistics 27. A pairwise Mann–Whitney U test was used to calculate the significance of untreated controls at different temperatures based on the ΔCT values of each gene with housekeeping *Act1*. Values at both higher temperatures were compared with values at 20–25 °C. Based on the values for the corrected significance, columns are highlighted with a star if the significance reaches a level of s. ≤ 0.001. For the evaluation of antifungal induction independent of the influence of temperature, ΔCT values of the induced gene expression of both temperatures were taken together, and a Kruskal–Wallis test was performed. Significances were corrected using the described Bonferroni formula. If induced probes differed from the untreated control at a level of s. < 0.001, columns were marked with a bracket and a star.

## 3. Results

The *T. indotineae* isolates UKJ 262/21 and UKJ 476/21 are *erg1*^Ala448Thr^ mutants showing differences in azole resistance patterns (Table S2 [15]). As an example, the 50% inhibitory concentrations (IC_50_ values) for itraconazole were approximately seven times lower for UKJ 262/21 compared to UKJ 476/21 (Table S2 [15]). Interestingly, UKJ 262/21 also belongs to the *erg11B*^Tyr444Cys^ mutants, whereas UKJ 476/21 carries the wild-type *Erg11B* gene variant (Table S2 [15]). Nevertheless, UKJ 476/21 exhibits the highest IC_50_ values for fluconazole, itraconazole and voriconazole compared to the isolates UKJ 262/21, CBS 146726, CBS 146727, UKJ 888/20 and UKJ 1067/21, which carry point mutations of *Erg11B* [15]. Gene expression analysis was performed to elucidate the molecular mechanism of the resistant phenotype found particularly in UKJ 476/21.

For the following study, gene expressions of squalene epoxidase gene *Erg1* and sterol 14-α demethylase genes *Erg11A* and *Erg11B* were analyzed to elucidate the activity of ergosterol biosynthesis. The influence on efflux mechanisms in fungal defense was determined by gene expression analysis of the multidrug resistance transporter genes *MDR1-3* and the major facilitator superfamily *MFS1* transporter. Due to the different growth temperatures during the experiment series, heat shock genes, specifically the transcription factor *HSF1* and the chaperones *HSP60* and *HSP90,* were further analyzed to evaluate the stress response of the isolates.

### 3.1. Multidrug Resistance Transporter MDR3 Expression Levels of T. indotineae Isolates Were Significantly Induced at Higher Temperatures or After Addition of Short-Chain Azoles

#### 3.1.1. Downregulation of MFS1 is Accompanied by Upregulation of MDR3 at Constant 37 °C in Terbinafine-Sensitive *T. indotineae* Isolates

Three series of experiments were carried out at different temperatures due to the fact that the room temperature differed significantly in the late summer months from that in autumn and winter. In summer, daily maximum temperatures of 37 °C were reached with periodic cooling at night. To partially replicate this, a second series was performed in a shaking water bath with a constant temperature of 37 °C. Finally, for a third series, the room temperature during autumn and winter was kept stable between 20 to 25 °C. Interestingly, the series at a constant 37 °C resulted in massive growth inhibition, particularly of the *erg1*^Leu393Ser^ mutant UKJ 888/20. Therefore, no RNA could be isolated. All other strains were able to grow under this condition without addition of antifungal compounds.

For a comparison of the expression levels of multiple *T. indotineae* strains, the values of all genes were normalized against isolate-specific *Act1* (actin) values as housekeeping control using the 2^−∆CT^ method [34].

**Figure 1:**
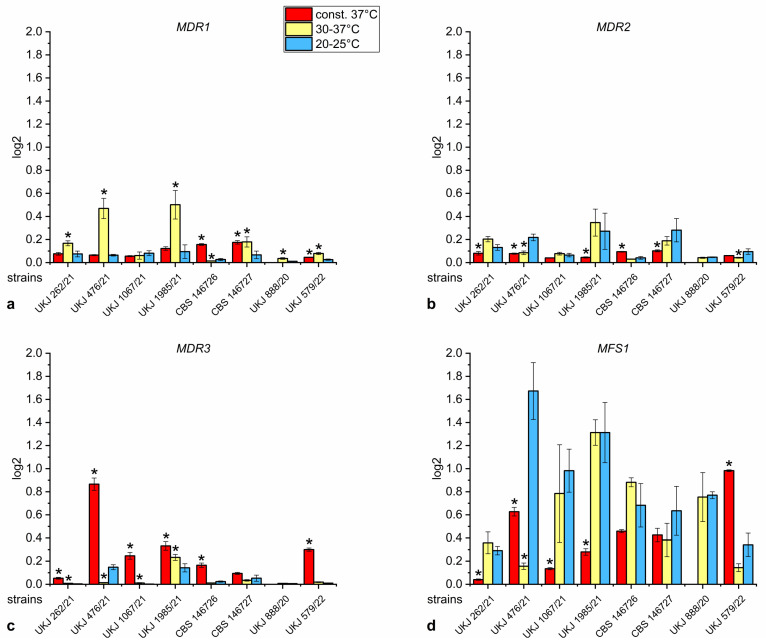
Relative expression of the transporter genes *MDR1* (**a**), *MDR2* (**b**), *MDR3* (**c**) or *MFS1* (**d**) of several *T. indotineae* isolates. Data were normalized to the housekeeper actin (*Act1*). Red columns show growth conditions at a constant 37 °C, yellow columns indicate periodical variations between 30–37 °C, and blue columns designate a growth temperature of 20–25 °C. A pairwise Mann–Whitney U test was performed with values of 20–25 °C as control. Columns marked with a star represent corrected significance values of s. ≤ 0.001.

The results of expression analyses for the transporter genes *MDR1*, *MDR2* and *MDR3* and the transporter of the major facilitator superfamily *MFS1* are shown in Figure 1a–d. High *MFS1* levels were observed in strains UKJ 476/21, UKJ 1067/21, UKJ 1985/21, CBS 146726 and UKJ 888/20 at temperatures of 20–25 °C (Figure 1d). The significant downregulation of *MFS1* at a constant 37 °C (Figure 1d) was associated with the essential upregulation of *MDR3* in several *T. indotineae* isolates (Figure 1c). Interestingly, significant changes were related to the terbinafine-sensitive group of *T. indotineae* isolates, including UKJ 262/21, UKJ 476/21, UKJ 1067/21 and UKJ 1985/21, as shown in Table S2 [15]. The strain UKJ 579/22 showed a unique pattern of increased *MFS1* and *MDR3* levels at a constant temperature of 37 °C (Figure 1c,d).

#### 3.1.2. Addition of Fluconazole Leads to Upregulation of MDR3 in the Majority of *T. indotineae* Isolates

**Figure 2:**
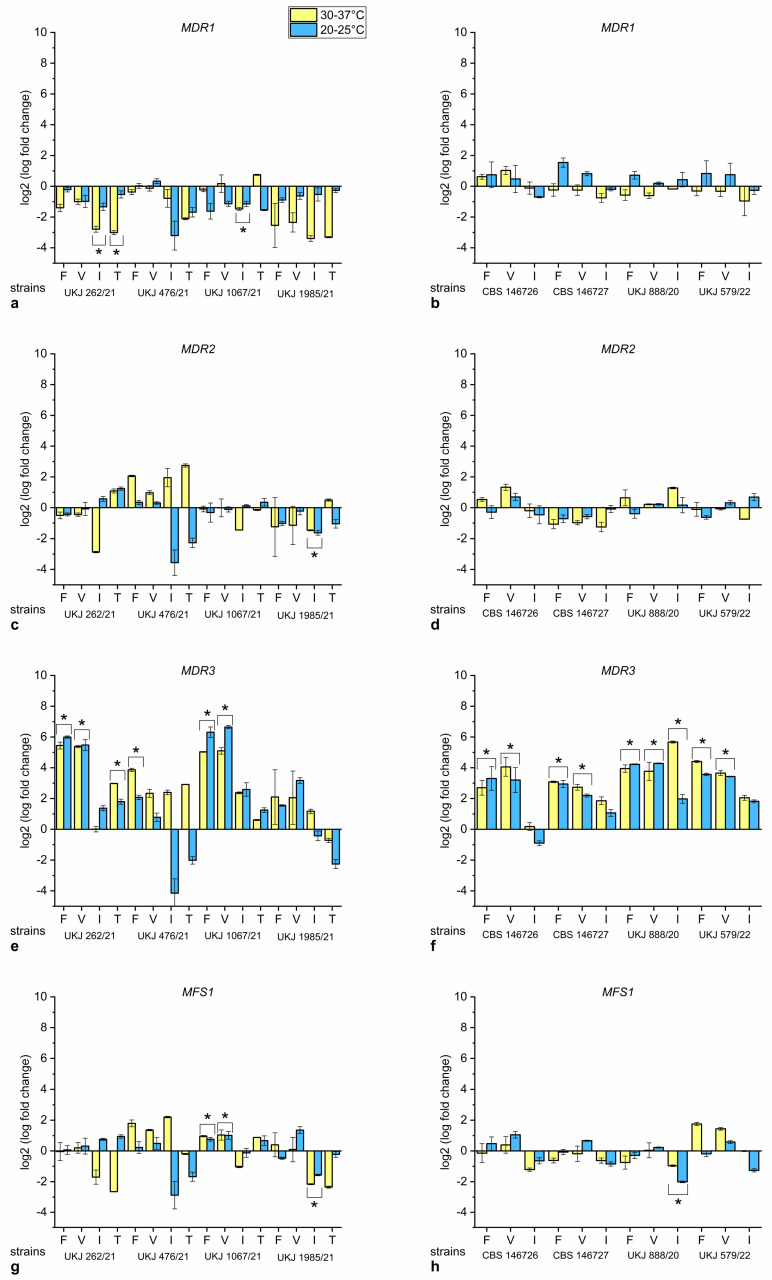
Expression fold change of *MDR1* (**a**,**b**), *MDR2* (**c**,**d**), *MDR3* (**e**,**f**) and *MFS1* (**g**,**h**) dependent on elected antifungal compounds. Values for terbinafine-sensitive *T. indotineae* isolates presented in (**a**,**c**,**e**,**g**) and those for terbinafine-resistant strains in (**b**,**d**,**f**,**g**). Fluconazole (F), voriconazole (V), itraconazole (I), or terbinafine (T) were added to the growth medium. Values of untreated controls were used for normalization. Values for both temperatures were analyzed together using statistical Kruskal–Wallis test parameters to evaluate significance related to antifungals additions. Marked columns with bracket and star show Bonferroni-corrected significance levels of s. < 0.001.

To measure the influence of antifungal compounds, fluconazole, voriconazole, itraconazole and terbinafine were added to the growth medium at concentrations below the expected fungistatic inhibition, based on the previously determined IC_50_ values [15]. The series at a constant 37 °C showed that the selected concentrations of antifungals in combination with the response to the permanent heat stress led to a strong growth inhibition in several *T. indotineae* isolates. Therefore, this dataset was incomplete and omitted from this analysis. The selected antifungal concentration range was appropriate for the other experimental series with temperature ranges of 30–37 °C and 20–25 °C. Fold-change expression levels were calculated from the values corrected for primer efficiency, as described [32]. The influence of terbinafine was only measured in strains susceptible to terbinafine. Fold-change data for terbinafine-sensitive strains are shown in in Figure 2a,c,e,g. The strains UKJ 262/21, UKJ 476/21 and UKJ 1067/21 showed upregulation of *MDR3* to significantly high levels if fluconazole was added to the medium (Figure 2e). Voriconazole demonstrated similar effects of increasing *MDR3* mRNA levels in some strains, as was for instance observed in isolates UKJ 262/21 and UKJ 1067/21 (Figure 2e). Terbinafine addition only led to a distinct upregulation of *MDR3* in UKJ 262/21 (Figure 2e). Downregulation of *MDR1* within the same strain also depended on terbinafine addition (Figure 2a). Itraconazole showed vital effects on *MDR1* downregulation, as observed in strains UKJ 262/21 and UKJ 1067/21 (Figure 2a), as well as on *MDR2* and *MFS1* expression decrease in UKJ 1985/21 (Figure 2c,g).

Interestingly, strain UKJ 1985/21, which was isolated from a relapse of the UKJ 1067/21-infected patient, showed a different expression pattern, especially for *MDR3*, compared to UKJ 1067/21 (Figure 2e). The induced effects of fluconazole and voriconazole on *MDR3* expression (Figure 2e) observed in UKJ 1067/21 were not detected in UKJ 1985/21. Moreover, the basic levels of *MDR3* expression were increased in UKJ 1985/21 compared to UKJ 1067/21 (Figure 1c).

In all terbinafine-resistant *T. indotineae* isolates, the addition of fluconazole or voriconazole resulted in a significantly increased expression of *MDR3* (Figure 2f). The effect of both short-chain azoles on *MDR3* expression was more pronounced in terbinafine-resistant strains and was to a lesser extent observed in terbinafine-sensitive strains (Figure 2f). No significant effects of antifungals on *MDR1* and *MDR2* gene expression were found (Figure 2b,d) for all terbinafine-resistant strains. Strain UKJ 888/20 demonstrated a unique pattern dependent on itraconazole addition, resulting in a distinct *MDR3* upregulation accompanied by a *MFS1* decrease (Figure 2f,h). In all other strains, independent of the terbinafine resistance status, itraconazole did not induce high levels of *MDR3* (Figure 2e,f). The *MFS1* expression levels of CBS 146726, CBS146727 and UKJ 579/22 showed no significant changes, irrespective of the addition of fluconazole, voriconazole or itraconazole.

### 3.2. Ergosterol Biosynthesis Gene Expression Is Influenced by Temperatures or Short-Chain Azoles

#### 3.2.1. Strain UKJ 476/21 Showed Exceptionally High Levels of Erg11B

**Figure 3:**
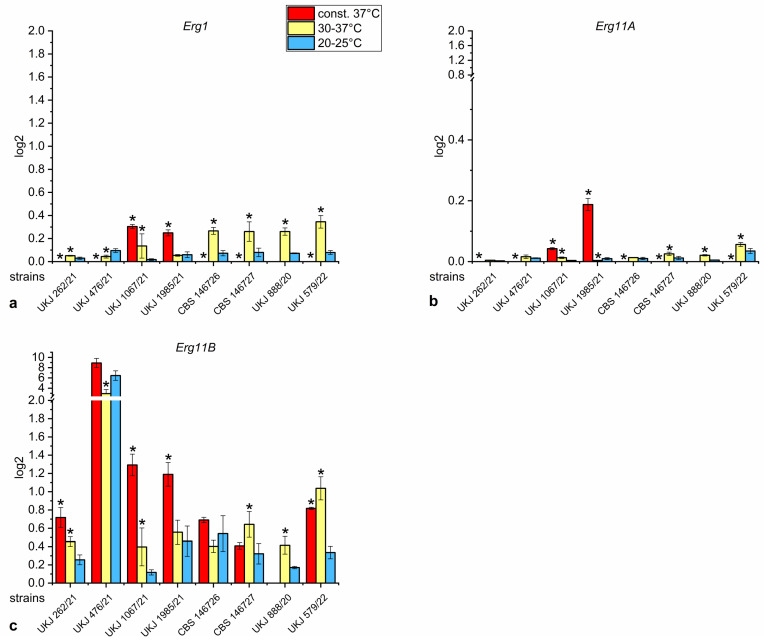
Relative expression of ergosterol biosynthesis genes *Erg1* (**a**), *Erg11A* (**b**) and *Erg11B* (**c**) of *T. indotineae* isolates. Data were normalized to the housekeeper actin (*Act1*). Red columns show growth conditions at a constant 37 °C, yellow columns indicate periodical variations between 30–37 °C, and blue columns designate a growth temperature of 20–25 °C. A pairwise Mann–Whitney U test was performed with values of 20–25 °C as the control. Columns marked with a star represent corrected significance values of s. ≤ 0.001.

When comparing all strains, the most prominent feature is the exceptionally high level of *Erg11B* transcripts in strain UKJ 476/21 regardless of the growth temperature condition (Figure 3c).

The *Erg1* expression pattern differed significantly in all temperature series. All terbinafine-resistant strains showed a significant increase in *Erg1* gene transcripts at 30–37 °C compared to 20–25 °C (Figure 3a). However, at a constant 37 °C, *Erg1* and *Erg11A* expression decreased significantly below relevant levels in the majority of strains (Figure 3a,b). This showed that the growth of these strains was distinctly impaired under these conditions. Only strains UKJ 1067/21 and UKJ 1985/21, isolated from the same patient, were an exception to this pattern. The two strains exhibited increased transcript levels for both genes at a constant 37 °C (Figure 3a,b, red columns), indicating a better adaptation to high growth temperatures. The expression pattern of *Erg1* and *Erg11A* for each strain showed a high similarity, which suggests the co-regulated transcription of both genes.

**Figure 4:**
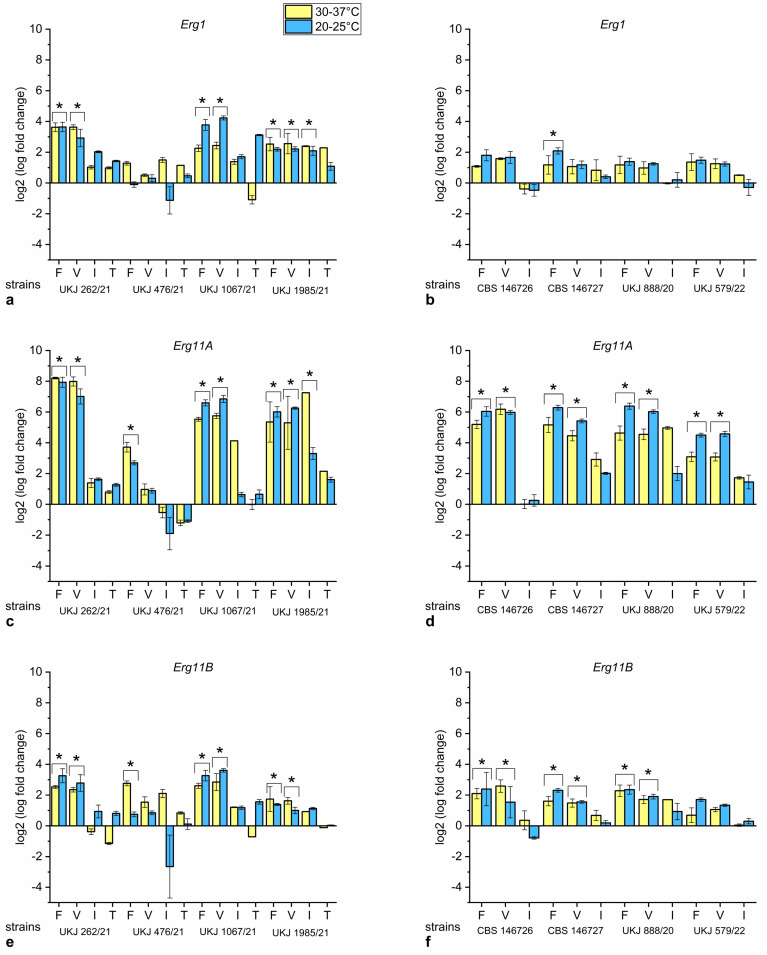
Expression fold change of *Erg1* (**a**,**b**), *Erg11A* (**c**,**d**) and *Erg11B* (**e**,**f**) dependent on selected antifungal compounds for *T. indotineae* isolates. Values for terbinafine-sensitive *T. indotineae* isolates presented in (**a**,**c**,**e**) and those for terbinafine-resistant strains in (**b**,**d**,**f**). Fluconazole (F), voriconazole (V), itraconazole (I), or terbinafine (T) were added to the growth medium. Values of untreated controls were used for normalization. Values for both temperatures were analyzed together using statistical Kruskal–Wallis test parameters to evaluate significance related to antifungals additions. Marked columns with bracket and star show Bonferroni-corrected significance levels of s. < 0.001.

Interestingly, high levels of *Erg11B* transcripts (Figure 3c, red columns) were found for UKJ 262/21, UKJ 476/21, CBS 146726, CBS 146727 and UKJ 579/22 at a constant 37 °C, even if *Erg1* and *Erg11A* expression was downregulated (Figure 4a,b). The unusual expression pattern of *Erg11B* raises questions regarding the role of Erg11Bp in de novo biosynthesis of ergosterol. The squalene epoxidase enzymatic function of Erg1p is necessary to produce substrates for the sterol 14-α demethylases Erg11p. Upregulation of *Erg11B* under conditions where fungal growth is impaired indicates a second role for Erg11Bp involvement in heat stress defense.

#### 3.2.2. Short-Chain Azoles Induce Transcriptional Expression of Erg11A

Strains UKJ 262/21, UKJ 1067/21 and UKJ 1987/21 showed a significant upregulation of *Erg1*, *Erg11A* and *Erg11B* in the presence of fluconazole or voriconazole (Figure 4a,c,e). The expression level of *Erg11A* demonstrated the highest fold-change values of all strains depending on fluconazole or voriconazole addition (Figure 4c,d). Only strain UKJ 476/21 was an exception to this pattern. This strain only showed a significant upregulation of *Erg11A* or *Erg11B* gene expression in response to fluconazole (Figure 4d,f). No significant changes were seen in *Erg1* expression for this strain (Figure 4b). In addition, *Erg11A* and *Erg11B* did not change during treatment with voriconazole, itraconazole or terbinafine (Figure 4c,e). The terbinafine-resistant group predominantly showed no distinct increase in *Erg1* mRNA independently of the antifungal used (Figure 4b). Significant effects under treatment with itraconazole were exclusively observed with UKJ 1985/21. In this strain, all tested azoles led to increased levels of *Erg11A* or *Erg1* mRNA transcripts (Figure 4a,c). Interestingly, terbinafine did not show a significant effect on *Erg1* expression in terbinafine-sensitive strains (Figure 4a). Although the enzymatic function of Erg1p is probably partially impaired due to the interaction with terbinafine, there is apparently no effective back loop mechanism to enhance *Erg1* transcription. Enhanced *Erg1* transcription is a possible defense mechanism for fungi against terbinafine. Short-chain azoles seem to be recognized in a different way compared to itraconazole or terbinafine and were able to induce transcription of all three genes.

### 3.3. Heat Shock Gene Expression Reacted to Increasing Temperature or Antifungals in a Competitive Manner

#### 3.3.1. Terbinafine-Resistant Strains Exhibited High Basal Heat Shock Gene Expression Levels

**Figure 5:**
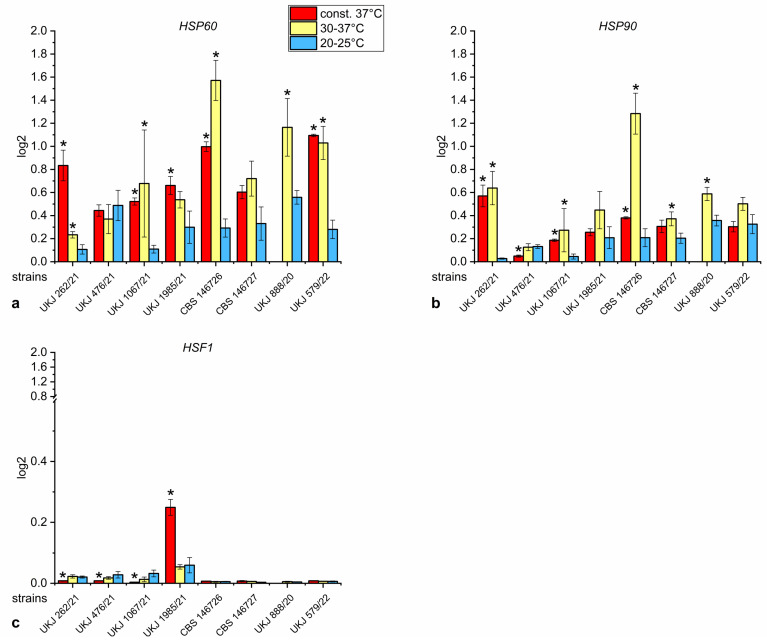
Relative expression of heat shock genes *HSP60* (**a**), *HSP90* (**b**) and *HSF1* (**c**) of *T. indotineae* isolates. Data were normalized to the housekeeper actin (*Act1*). Red columns show growth conditions at a constant 37 °C, yellow columns indicate periodical variations between 30–37 °C, and blue columns designate a growth temperature of 20–25 °C. A pairwise Mann–Whitney U test was performed with values of 20–25 °C as the control. Columns marked with a star represent corrected significance values of s. ≤ 0.001.

High gene transcript levels of the chaperones *HSP60* and *HSP90* were found at higher temperatures for UKJ 262/21, UKJ 1067/21, CBS 146726 and UKJ 888/20 (Figure 5a,b). For UKJ 1985/21 and UKJ 579/21, significantly increased levels were solely observed for *HSP60* (Figure 5a).

**Figure 6:**
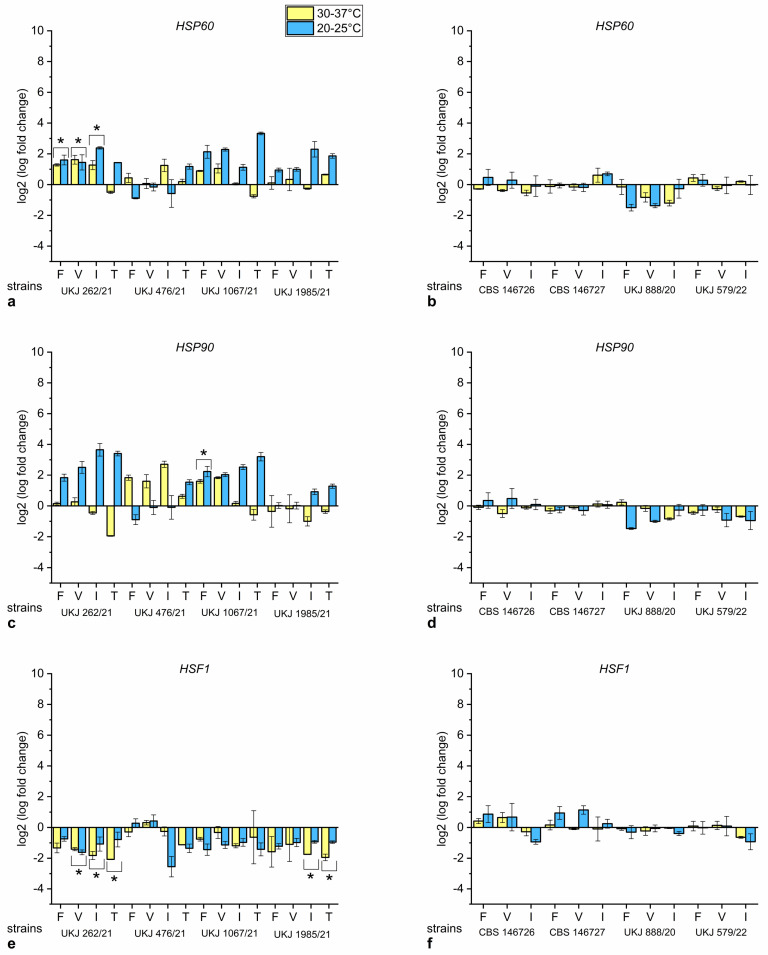
Expression fold change of *HSP60* (**a**,**b**), *HSP90* (**c**,**d**) and *HSF1* (**e**,**f**) dependent on selected antifungal compounds for *T. indotineae* isolates. Values for terbinafine-sensitive *T. indotineae* isolates presented in (**a**,**c,e**) and those for terbinafine-resistant strains in (**b**,**d**,**f**). Fluconazole (F), voriconazole (V), itraconazole (I), or terbinafine (T) were added to the growth medium. Values of untreated controls were used for normalization. Values for both temperatures were analyzed together using statistical Kruskal–Wallis test parameters to evaluate significance related to antifungals additions. Marked columns with bracket and star showed Bonferroni-corrected significance levels of s. < 0.001.

The expression pattern of UKJ 476/21 showed a unique feature: the heat shock gene expression of the chaperones *HSP60* and *HSP90* did not respond to increasing temperatures, as was the case for all other analyzed *T. indotineae* isolates (Figure 5a,b). At a constant 37 °C, *HSP90* was significantly downregulated compared to the two other experimental series with lower temperatures, whereas in all other isolates, upregulation or steady-state levels of *HSP90* expression were observed (Figure 5b). Moreover, even at low temperatures, high basal levels of *HSP60* were detected in UKJ 476/21 (Figure 6a), suggesting that an unknown signal activated the stress response.

Interestingly, strains UKJ 262/21 and UKJ 1067/21 showed a low basic expression of *HSP60* and *HSP90* at 20–25 °C when compared to all other isolates (Figure 5a,b). These two strains belong to the terbinafine-sensitive group. In contrast, all terbinafine-resistant strains already expressed high basal levels of *HSP60* and *HSP90* at the low-temperature condition, which was accompanied by further upregulation at increasing temperatures (Figure 5a,b). Moreover, the basal *HSP60* and *HSP90* expression of UKJ 888/20 started at a high level at this temperature (Figure 5a,b), indicating that even at low temperatures the stress-dependent reaction is activated due to unknown signals. This strain showed the highest sensitivity to elevated growth temperatures.

All terbinafine-resistant isolates exhibited low expression levels of the major transcription factor *HSF1*, responsible for the regulation of proteostasis (Figure 6c). Terbinafine-sensitive strains showed higher basal levels of *HSF1* expression at 20–25 °C as well as at 30–37 °C (Figure 5c) in comparison to the resistant strains. At a constant 37 °C, a significant downregulation of *HFS1* was observed in UKJ 262/21, UKJ476/21 and UKJ 1067/21 (Figure 5c). In contrast, UKJ 1985/21 showed distinctly increased *HFS1* expression levels at a constant 37 °C (Figure 5c). UKJ 1985/21 demonstrated a better growth adaptation to higher temperatures, and ergosterol biosynthesis genes *Erg1* and *Erg11A* were also upregulated under this condition (Figure 3a,b).

#### 3.3.2. Temperature and Antifungals Often Acted Antagonistically on Heat Shock Gene Expression

In UKJ 262/21, all antifungal compounds upregulated the heat shock protein gene expression of *HSP90* at low temperatures (Figure 6c). In UKJ 1067/21 and UKJ 1985/21, itraconazole or terbinafine produced a similar effect, with the levels being higher for UKJ 1067/21 (Figure 6c). Interestingly, the basal level of *HSP90* was increased in UKJ 1985/21 compared to UKJ 262/21 and UKJ 1067/21 (Figure 5b). *HSP60* expression was induced at increasing temperatures in UKJ 262/21 when azoles were added (Figure 6a). In UKJ 1067/21 and UKJ 1985/21, upregulation of *HSP60* for all added compounds was only observed at low temperatures (Figure 6a). Significant upregulation of *HSP90* at both temperatures was solely found in UKJ 1067/21 under treatment with fluconazole.

UKJ 476/21 exhibited increased *HSP90* levels at higher temperatures, with the highest fold changes found after itraconazole treatment (Figure 6c). At low temperatures, the azoles did not lead to gene expression changes in UKJ 476/21 (Figure 6c). This strain did not exhibit a heat-dependent increase of *HSP60* and *HSP90* expression levels without antifungal treatment (Figure 5a,b). If the heat shock gene was expressed at high basal levels, further stimulation failed as it may be more difficult to detect additional stress signals. Therefore, antifungal-dependent stimulation of *HSP60* and *HSP90* expression was observed predominantly in UKJ 262/21 and UKJ 1067/21 (Figure 6a,c).

No terbinafine-resistant strain responded to antifungals, with differences in the expression of all three analyzed heat shock genes (Figure 6b,d,f). Nevertheless, upregulation of *HSP60* and *HSP90* expression at increasing temperatures was observed for all strains of this group (Figure 5a,b). *HSF1* expression started at low levels and did not demonstrate a heat-dependent response within this group (Figure 5c). Significant *HSF1* downregulation was observed in UKJ 262/21 and UKJ 1985/21 (Figure 6e). All sensitive strains started at higher basal expression levels of *HSF1,* and UKJ 1985/21 showed the highest values for *HSF1* (Figure 5c).

The input of several signals was able to modify heat shock gene expression and act in a competitive manner. The activation of chaperones at low temperatures in the terbinafine-resistant isolates may be the reaction to Erg1p with amino acid exchanges at positions 393 or 397 that is recognized as a protein misfold.

## 4. Discussion

The *erg1*^Ala448Thr^ strain UKJ 476/21-derived expression pattern was found to be different from all other *T. indotinae* isolates investigated. In particular, the basal level of *Erg11B* transcription is upregulated to an exceptionally high level (Figure 3c). The expression pattern corresponds to the increased tolerance of UKJ 476/21 to clinically used azoles, such as fluconazole, voriconazole or itraconazole [15]. Overexpression of *Erg11B* was also found in other *T. indotineae* isolates, which correlated with increased *Erg11B* copy numbers [18]. Nevertheless, strain TIMM 20,117 also showed an increase in *Erg11B* transcription, although genome analysis reveals the single copy status of *Erg11B* [18]. Interestingly, this strain belongs to *erg1*^Ala448Thr^ mutants such as UKJ 476/21. An interesting question is why the transcriptional regulators of *Erg11B* do not counteract the increased *Erg11B* copy numbers and why no feedback response was observed.

The differences in the expression patterns of both *Erg11* genes may reflect different roles in the signaling pathways. *Saccharomyces cerevisiae* and *Candia albicans* harbor only one *Erg11* copy, whereas *Aspergillus fumigatus* has two copies of *Erg11*, designated A and B. Lanosterol is the natural substrate of yeast *Erg11*, although other substrates, such as eburicol (24-methylene-dihydrolanosterol), have also been accepted in vitro [35]. In *Aspergillus fumigatus*, the two Erg11 enzymes differ in substrate specificity, and *A. fumigatus* Erg11Bp only converts eburicol without the ability to metabolize lanosterol [36]. Erg11Ap differs in the substrate cavity and may also use lanosterol as a substrate [36]. The predicted amino acid differences of the substrate binding cavity [36] were also found in *Erg11A* and *Erg11B* of *T. indotineae*. Therefore, *T. indotineae Erg11B* more closely resembles the *A. fumigatus* eburicol demethylase Erg11B. Eburicol is produced by the enzymatic action of *Erg6* using lanosterol as a substrate [35,37]. Deletion mutants of *A. fumigatus Erg11B* accumulate C-4- and C-14 methyl sterols with eburicol as the main component [38]. In *Aspergillus nidulans,* any deletion mutant of *Erg11A* or *B* is viable, and only double mutants result in a lethal phenotype [37]. Interestingly, eburicol is mentioned as a toxic sterol when it accumulates in *A. fumigatus,* which explains the fungitoxic properties of azoles in this fungus [38]. If eburicol has similar toxic effects in *T. indotineae*, a second function of *Erg11B* could be the detoxification of eburicol. Azole resistance mechanisms in *A. fumigatus* depended in general on the overexpression of *Erg11A* or of those *Erg11A* point mutations that lead to amino acid exchanges [37,39]. In *T. indotineae,* overexpression of *Erg11B* [18] as well as *Erg11B* point mutations were detected, whereas no genetic variability of *Erg11A* was observed [15]. Interestingly, *Erg11A* mutants of *Trichophyton quinckeanum*, a mice pathogen causing favus, demonstrated increased tolerance to itraconazole [19]. *T. indotineae Erg11B* point mutations [15] affect a fungal specific loop that forms an additional β-bundle from two adjacent antiparallel β-strands (β5-1 and β5-2) [36]. This fungus-specific β5-bundle is located between two conserved elements called meander and heme bulge [36]. In *A. fumigatus,* the region may be involved in the electron transfer process and interaction with P450 reductase, modeling the heme environment [36]. *T. indotineae Erg11B* mutants at amino acid position 444, such as UKJ 262/21, CBS 146726 or UKJ 1067/21, exhibited sensitivity to all azoles typically used in medical treatments [15]. The *Erg11B* mutants UKJ 888/20 and CBS 146727 showed a fourfold higher tolerance to itraconazole [15] and demonstrated an itraconazole-induced expression pattern of *MDR3* and *Erg11A* (Figure 3). Therefore, these strains activate efflux of azoles and compensate Erg11 inhibition with increased levels of Erg11A proteins.

Interestingly, *Erg11* mutants of the phytopathogenic fungus *Mycosphaerella graminicola* (syn. *Zymoseptoria tritici*) comprise mutations at comparable positions [40], as detected in *T. indotineae Erg11B.* In *Mycosphaerella fijiensis,* similar mutations lead to resistance to propiconazole [41]. Phylogenetic analyses of strains of the *T. mentagrophytes*/*interdigitale* complex showed the basal lineage of *T. indotineae* [2,4]. So far, an animal reservoir for *T. indotineae* was not detected. Nevertheless, the near relation to zoophilic strains of the *T. mentagrophytes/interdigitale* complex and inflammatory infection pattern in humans enhance the probability of an animal reservoir. Azole resistance may have developed in zoophilic isolates of a wild animal reservoir, if this had come into contact with azoles used in agriculture, or it may have started in *T. indotineae* isolates of infected farmers using azoles in their fieldwork. This could be an explanation for why *T. indotineae* mutations in *Erg11B* resemble those of phytopathogenic fungi.

In *Aspergillus fumigatus*, the azole resistance of non-clinical isolates, obtained from the environment, often demonstrates overexpression of *Erg11A* due to changes in the promoter region [42]. This leads to a more general azole resistance mechanism and results in multiple resistances against medically used azoles [42]. A similar phenomenon is observed in *T. indotineae* isolates that exhibit overexpression of *Erg11B.* Multiple azole resistances, as described for UKJ 476/21 [15], can be explained by the overexpression pattern of *Erg11B* shown in this study. The increase in *T. indotineae Erg11B* copy numbers is also accompanied by an overexpression pattern and can further explain the phenotype of azole resistance [18].

Therefore, the usage of azoles in agriculture and cross-resistance with medicinal azoles pose a serious problem to human health.

## 5. Conclusions

Overexpression of *Erg11B* explains the multidrug-resistant phenotype of UKJ 476/21. Heat-dependent *Erg11B* expression in the absence of *Erg1* expression suggests a secondary role for *Erg11B* in the detoxification of putatively toxic methyl-sterols. The expression of *MDR3* and *Erg11A* increased in the presence of fluconazole or voriconazole, whereas itraconazole did not stimulate expression in several strains. The strain UKJ 1985/21, isolated from a relapse, showed persistently increased levels of *MDR* transporters and heat shock proteins without further stimulation.

The zoonotic character of several *Trichophyton* species makes it necessary to increase the surveillance of resistance mechanisms, even in zoophilic species. Azoles in agriculture have the potential to increase the selection of azole-resistant fungal pathogens of wild animals. It is a small step for a pathogen such as *Trichophyton* to change the host preference and has been observed several times in the past. The *Allylamines* resistances of fungal pathogens were the result of medical treatments without enough surveillance of the resistance mechanisms of the pathogen. This class of substances was used only for medical treatment. It is necessary to increase surveillance, especially for patients who need a long-term treatment to cure dermatomycosis.

## Data Availability

DNA sequence data were stored in GenBank with Acc. No. PP537547-PP537548 and GenBank Acc. No. PP549424-PP549429.

## Supplementary Materials

The following supporting information can be downloaded at: https://www.mdpi.com/article/10.3390/jof10110731/s1, Table S1: GenBank Acc. Nos. of sequenced DNA fragments.; Table S2: *Erg1* and *Erg11B* mutations of *T. indotineae* strains and their resistance patterns. Table S3: Adjusted primer list for quantitative real-time PCR of *T. indotineae* fragments.
