# Supplementary material for: Trichophyton indotineae Erg1Ala448Thr Strain Expressed Constitutively High Levels of Sterol 14-α Demethylase Erg11B mRNA, While Transporter MDR3 and Erg11A mRNA Expression Was Induced After Addition of Short Chain Azoles
Source: J Fungi (Basel). 2024 Oct 22;10(11):731. doi: 10.3390/jof10110731 (PMC11595479; doi:10.3390/jof10110731)
Supplement: Supplementary file 1 [file jof-10-00731-s001.zip › jof-3218962-supplementary.pdf]

Trichophyton indotineae Erg1$^{\mathrm{Ala448Thr}}$ Strain Expressed Constitutively High Levels of Sterol 14-α Demethylase *Erg11B* mRNA and *Erg11A* and Transporter *MDR3* Expression Induced After Addition of Short Chain azoles in Majority of Other Isolates

Nadine Berstecher, Anke Burmester, Deborah M. Gregersen, Jörg Tittelbach and Cornelia Wiegand

Supplementary Materials

Table S1. GenBank Acc. No. of sequenced DNA fragments.

Species	Strain GenBank	*Erg1* Acc. No.	*Erg11A* Acc. No.	*Erg11B* Acc. No.	*ITS* Acc. No. *
T. indotineae	CBS146726	MN068042	MZ636367	MZ636372	MN0648226
	CBS146727	MT700528	MZ636368	MZ636373	MZ647518
	UKJ888/20	MZ636377	MZ636369	MZ636374	MZ614625
	UKJ262/21	MZ636378	MZ636371	MZ636376	MZ614626
	UKJ476/21	MZ636379	MZ636370	MZ636375	MZ614627
	UKJ1067/21	OK572480	OK572478	OK572479	OK569897
	UKJ1985/21	PP549428	PP549424	PP549426	PP537547
	UKJ579/22	PP549429	PP549425	PP549427	PP537548

* GenBank Acc. No. starting with MN were as mentioned previously [7], with MT as cited [16], with MZ, OK as in [15] and with PP this work.

Table S2. *Erg1* and *Erg11B* mutations of *T. indotineae* strains and their resistance patterns. All strains showed wild type *Erg11A* gene sequences. *Erg*1 mutant strains at amino acid position 397 and 393 showed highly increased level of inhibitory concentrations (IC) against terbinafine [15] and represent the terbinafine resistant group of *T. indotineae* strains.

Species	Strain *	*Erg1* Mutations	*Erg11B* Mutations	Terbinafine IC_{50} µg/mL	Itraconazole IC_{50} µg/mL
T. indotineae	CBS146726	Phe397Leu	Tyr444His	2.7	0.0015
	CBS146727	Phe397Leu	Ala230Thr<br>Asp441Gly	2.3	0.0079
	UKJ888/20	Leu393Ser	Gly443Glu	0.16	0.0088
	UKJ262/21	Ala448Thr	Tyr444Cys	0.0043	0.0022
	UKJ476/21	Ala448Thr	Wild type	0.0038	0.015
	UKJ1067/21	Wild type	Ala230Thr<br>Tyr444His	0.0089	0.0027
	UKJ1985/21	Wild type	Ala230Thr<br>Tyr444His	0.013	0.0024
	UKJ579/22	Phe397Leu	Asp441Gly	2.8	0.0012

* Strains CBS 14626 up to UKJ 1067/21 were as mentioned previously [15], UKJ 1985/21 and UKJ 579/22 data obtained for this work.

Table S3. Adjusted primer list for quantitative real-time PCR of *T. indotineae* fragments.

Gene	Primer Name	Sequence 5′3′
MDR1 [1]	TrMDR1for1	CCTAATGCCCTTCCTGGATT
	TrMDR1rev1	AAATTGCCAGCTCGTTCTGT
MDR2 [1]	TmMDR2for2	CGACTCTGAATCCGAAAAGG
	TrMDR2rev1	GTCGGTGAGCAACAGCAATA
MDR3 [1]	TrMDR3for1	TCACTGGTGAAATGCTCGTTGACG
	TmMDR3rev2	GGTTGACGGAGCAAGGCGCTG
MFS1 [1]	TrMFS1for1	ATTATCGGCCGTGCAGTTGC
	TrMFS1rev1	ACCTGCCACAGAGGCAATACC
ACT1 [1]	TrACT1for1	TGTTGGTGATGAGGCACAGT
	TrACT1rev1	CCATGTCATCCCAGTTTGTG
Erg1 [2]	TmErg1for2	CCAGACTGATGGCAAGCAAGA
	TrErg1rev1	ATAAGCTCCAGGCCCCAGAA
Erg11A	TmErg11Afor1	AGGATGTGAATGCAGAGGAG
	TmErg11Arev1	CGATCAATGGAACGTAAGCC
Erg11B	TmErg11Bfor1	TCCCAACCTCTCACAACCTC
	TmErg11Brev1	CACCCTTGCTAACCAAACCG
HSF1 [3]	TmHSF1for2	GTGCTGGAGGCCGAGAAG
	TrHSF1rev1	TCCCGACCCGAGAGCAA
HSP60 [3]	TrHSP60for1	AAGCGTCGTTGTCGGTAAGC
	TmHSP60for2	TGTCGAAGCCACGGTTGAAGT
HSP90 [3]	TrHSP90for1	ACCGTGCTGCCCTTGCT
	TmHSP90rev2	GTGATCTCATCACCAGATTTG

[1] Primer sequences derived as previously described [21], [2] as published in [26] and presented here [3] [27] . Modification was included if *T. indotineae* genome information of D15P135 [4] show DNA sequence differences.
